# The association of gout with sleep disorders: a cross-sectional study in primary care

**DOI:** 10.1186/1471-2474-14-119

**Published:** 2013-04-04

**Authors:** Edward Roddy, Sara Muller, Richard Hayward, Christian D Mallen

**Affiliations:** 1 Arthritis Research UK Primary Care Centre, Keele University, Keele, UK

**Keywords:** Gout, Sleep, Apnea, General practice, Metabolic syndrome X

## Abstract

**Background:**

Both gout and sleep apnoea are associated with the metabolic syndrome. Hyperuricaemia is also prevalent in sleep apnoea syndrome. The objective of this study was to examine the association between gout and sleep apnoea and other sleep disorders.

**Methods:**

Data were taken from a validated database of general practice records from nine practices in the UK between 2001 and 2008. People consulting for gout were identified via Read codes and each matched with four controls for age, gender, practice and year of gout consultation. Sleep problems and confounding comorbidities were also identified via Read codes. Medications were identified through a linked database of prescription records. The association between gout and sleep disorders was assessed using a logistic regression model, adjusting for ischaemic heart disease, hypertension, diabetes mellitus and diuretic use.

**Results:**

1689 individuals with gout were identified and each successfully matched to four controls. Amongst those with gout, the prevalence of any sleep problem was 4.9%, sleep problems other than sleep apnoea 4.2%, and sleep apnoea 0.7%, compared to 3.5%, 3.2% and 0.3% respectively in controls. Gout was associated with any sleep problem (odds ratio (OR) 1.44; 95% confidence interval (CI) 1.11, 1.87), sleep problems other than sleep apnoea (OR 1.36; 95% CI 1.03, 1.80), and sleep apnoea (OR 2.10; 95% CI 1.01, 4.39). On multivariable analysis, gout remained significantly associated with any sleep problem (OR 1.39; 95% CI 1.06, 1.81) and sleep problems other than sleep apnoea (OR 1.37; 95% CI 1.03, 1.82), however the association with sleep apnoea was attenuated (OR 1.48, 95% CI 0.70, 3.14).

**Conclusions:**

Gout and sleep problems appear to be associated and clinicians should be aware of the co-existence of these two conditions. Larger prospective epidemiological studies are required to explore causality.

## Background

Gout is the most prevalent inflammatory arthropathy and affects approximately 1.4% of adults [1,2]. It is associated with considerable co-morbidity. The metabolic syndrome is present in over 60% of individuals with gout, who are more than three times more likely to have the metabolic syndrome than control subjects without gout [3]. Individual components of the metabolic syndrome such as hypertension, obesity and diabetes mellitus are recognised to be independent risk factors for the development of gout [4]. Insulin resistance is thought to be the major mediator of hyperuricaemia, the primary risk factor for the development of gout, in the metabolic syndrome [5] although hypertension exerts renal vascular effects which also predispose to hyperuricaemia [6]. Perhaps, not surprisingly, given its association with traditional cardiovascular risk factors, gout is associated with a considerable burden of cardiovascular disease. However, recent prospective epidemiological studies have suggested that gout confers additional independent cardiovascular risk after adjustment for traditional risk factors [7-10].

Obstructive sleep apnoea syndrome is also a common problem in primary care having a similar prevalence to gout of approximately 1–4% [11,12], although there is evidence that it is under-diagnosed in this setting [13,14]. Similar to gout, obstructive sleep apnoea syndrome is associated with the metabolic syndrome, obesity, hypertension, hyperlipidaemia, diabetes mellitus and cardiovascular disease [15-21]. It is less widely recognised that serum uric acid levels are also frequently elevated in patients with obstructive sleep apnoea syndrome. One-quarter to one-half of patients with obstructive sleep apnoea syndrome have been shown to have hyperuricaemia [19,22-24]. However, despite prevalent hyperuricaemia in patients with obstructive sleep apnoea syndrome and shared risk factors with gout of obesity and alcohol consumption [25,26], little consideration appears to have been given to the possibility of an association between obstructive sleep apnoea syndrome and gout [27]. A biologically plausible mechanism exists to explain hyperuricaemia in obstructive sleep apnoea syndrome. Hypoxia enhances nucleotide turnover [28-30] generating purines which are metabolised to uric acid.

We undertook a cross-sectional study within a regional primary care consultation database with the aims of, firstly, determining whether an association exists between gout and obstructive sleep apnoea syndrome and other sleep disorders and, secondly, whether any such association is independent of co-morbid traditional vascular risk factors and vascular disease.

## Methods

### Study Setting

The data for this study are taken from two inter-linked primary care databases: the Consultations in Primary Care Archive (CiPCA) and the Prescriptions in Primary Care Archive (PiPCA). These databases hold records of all consultations and prescriptions of patients at 13 general practices in North Staffordshire, UK, and have been extensively described elsewhere [31]. The practices form part of the Keele General Practice Research Partnership, and regularly undergo cycles of training, assessment and feedback regarding the quality of computerised morbidity coding [32]. Comparable consultation rates have been shown in CiPCA, as in national databases such as the General Practice Research Database [31].

Practice staff are trained to enter at least one Read code relating to each patient consultation. The Read code classification is a hierarchy of morbidity, symptom and process codes, organised by anatomical symptom, that become more specific the lower down the coding hierarchy one progresses [33].

All prescriptions issued at the participating practices are automatically coded according to the British National Formulary (BNF) and entered into the PiPCA database. This ensures complete ascertainment of all prescriptions issued.

All patients are allocated a unique identifier, allowing their records to be linked over time and between the CiPCA and PiPCA databases. For this study, data from nine of the participating practices, who provided data to the CiPCA and PiPCA databases for the complete period from 2001 to 2008 and used version five of the Read code system, were included.

### Cases

Patients consulting their general practice and receiving a Read code for gout between 2001 and 2008 were identified in the CiPCA database. A full list of the codes used to identify cases is available from the authors on request. We have previously examined the clinical records within the CiPCA database of those people who received a Read code for gout and found features of inflammation and distribution of affected joints consistent with a diagnosis of gout [34]. The full consultation and prescription record from 2001 to 2008 was downloaded for each of these patients.

### Controls

Controls were matched 4:1 to cases within the CiPCA database. Controls were randomly selected using frequency matching for age (within three-year bands), gender and general practice. Controls had also consulted their family physician for any non-gout condition within the same calendar year as the first gout consultation after 2001 was identified in cases. As with controls, all consultations and prescriptions from 2001 to 2008 were downloaded for these individuals.

### Sleep disorders

Sleep disorders were identified in the medical records of all cases and controls using Read codes. Specific Read codes used in this search are available from the authors on request. From these Read codes, each individual was categorised as either having a sleep disorder recorded at some time during the study period (2001 to 2008) or not. Within those with a record of a sleep disorder, those who specifically received a code for sleep apnoea were identified and classified as having sleep apnoea. Those with a Read code for a sleep disorder, but no code for sleep apnoea were classified as having sleep problems other than sleep apnoea; forming two mutually exclusive groups.

### Comorbid confounders

Type II diabetes mellitus, ischaemic heart disease (IHD), hypertension, and use of diuretic drugs were considered potential confounders in the association between gout and sleep disorders. Read codes were used to identify those cases and controls with type II diabetes mellitus, IHD and hypertension within the 2001 to 2008 period of interest. People with any prescriptions for diuretics between 2001 and 2008 were identified from the PiPCA database using British National Formulary (BNF) codes. Read and BNF codes used to identify potential confounders are available from the authors on request.

### Statistical analyses

The proportion of people with recorded sleep disorders was described. Differences in the proportion of people with these disorders in the cases and controls were compared using confidence intervals, calculated in the program Confidence Interval Analysis [35].

In order to assess the extent of clustering of patients within general practices, a multi-level model with no independent variables was fitted. Based on the findings of this model, multi- or single-level logistic regression models were fitted as appropriate to assess the association between gout and the presence of sleep disorders. Adjustment was made for the matching factors of age, gender and year of consultation. Fractional polynomials were used to assess the linearity of any association with age and year of first consultation. Analyses were adjusted for the potentially confounding comorbidities as described above. Regression analyses were conducted in Stata 11.2. The -mfp- command was used to estimate fractional polynomials [36].

### Ethical approval

The CiPCA database has ethical approval for case–control studies from the North Staffordshire local research ethics committee (approval number 03/04). This approval did not require individual informed consent, as data were analysed anonymously.

## Results

### Case and control selection

Within the CiPCA database, 1689 people were identified as having consulted their GP for gout between 2001 and 2008. Cases were successfully matched to controls (n = 6756) in a 1:4 ratio, as described above. Cases were well-matched to controls in terms of the matching variables (Table 1). The prevalence of hypertension, IHD and type II diabetes mellitus was higher among gout cases than controls, as was the prescription of diuretic medications.

**Table 1:** Comparison of gout cases (n = 1689) and controls (n = 6756) from the CiPCA dataset

**Characteristic**	**Case (n (%))**	**Control (n (%))**
Age (mean (SD))	62.5 (15.3)	62.5 (15.4)
Female gender	397 (23.5)	1588 (23.5)
Type II diabetes mellitus	212 (12.6)	615 (9.1)
Ischaemic heart disease	348 (20.6)	1065 (15.8)
Hypertension	919 (54.4)	2516 (37.2)
Diuretic prescription	903 (53.5)	2411 (35.7)

### Association between gout and sleep disorders

Overall, 3.7% (n = 316) of those studied had at least one Read coded consultation for a sleep disorder, of which 10.1% (n = 32) had a Read code for sleep apnoea. The proportion of people with any sleep disorder was higher in the gout cases (4.9%) than in the controls (3.5%) (1.4% difference, 95% CI: 0.3%, 2.5%) (Table 2). There was no effect of clustering by general practice. Hence, single level logistic regression models, adjusted for practice, were used for all analyses. After adjustment for potentially confounding comorbidities and diuretic prescriptions, participants continued to have 1.4 times the odds of gout if they had a sleep disorder than if they did not (OR 1.39; 95% CI 1.06, 1.81).

**Table 2:** Association of sleep problems and gout: results of logistic regression analyses

	**Cases**	**Controls**	**Unadjusted**^**a**^**OR (95% CI)**	**Adjusted OR**^**b**^**(95% CI)**
Any sleep problem	82 (4.9)	234 (3.5)	1.44 (1.11, 1.87)	1.39 (1.06, 1.81)
Sleep apnoea	11 (0.7)	21 (0.3)	2.10 (1.01, 4.39)	1.49 (0.70, 3.14)
Sleep problems other than sleep apnoea	71 (4.2)	213 (3.2)	1.36 (1.03, 1.80)	1.37 (1.03, 1.82)

OR, odds ratio.

For both models, age and year of first gout consultation are fitted linearly. ^a^Adjustment made only for matching variables (age, year of first gout consultation, gender, practice). ^b^Adjustment made for matching variables and potentially confounding factors (type II diabetes mellitus, ischaemic heart disease, hypertension and diuretic prescription).

There was a 1.4-fold increased odds of gout in those with a sleep disorder but no record of sleep apnoea (OR 1.36; 95% CI 1.03, 1.80). After adjustment for potential confounders, this association remained significant (OR1.37; 95% CI 1.03, 1.82). Sleep apnoea was also coded in a higher proportion of gout cases than controls (0.7% of cases and 0.3% of controls). This amounted to a two-fold increased odds of gout in those with sleep apnoea than in those without (OR 2.1; 95% CI 1.01, 4.39). However, after adjustment for potentially confounding comorbidities and medication prescriptions, this increase in odds was attenuated 1.49 (95% CI 0.70, 3.14).

## Discussion

To our knowledge, apart from one previous case report [27], this is the first empirical epidemiological study to explore an association between gout and obstructive sleep apnoea syndrome. We examined the association between gout and firstly all sleep problems combined (“any sleep problem”) and secondly sleep problems re-categorised as sleep apnoea and sleep problems other than sleep apnoea. There were small but significant associations between gout and both any sleep problem and sleep problems other than sleep apnoea which remained largely unchanged after adjusting for associated co-morbid conditions. Although we found a two-fold increased risk of sleep apnoea in those with gout on univariable analysis, this was no longer statistically significant after adjustment for multiple confounding variables.

The strengths of the study are its primary care setting and the rigorous quality assurance processes undertaken to ensure the robustness of computerized morbidity coding in the participating practices [32]. Limitations of our study are worthy of acknowledgement. First, the cross-sectional study design merely informs about association and does not allow for any inferences concerning causality or direction of association. Second, use of primary care consultation data relies upon a primary care diagnosis which may introduce misdiagnosis and hence misclassification bias. In primary care, the diagnosis of gout is most frequently made on clinical grounds [37,38] whereas definitive diagnosis requires crystal identification [39]. It has previously been suggested that gout is frequently over-diagnosed in primary care [40]. However, primary care consultation records have been widely used in gout research [1,2,41,42] and, although not undertaken during the current analysis, in a previous study undertaken using consultation data from the CiPCA database, scrutiny of consultation free text recorded by general practitioners confirmed features of inflammation and distribution of affected joints generally consistent with a diagnosis of gout [34]. Diagnosis in primary care is also an important consideration for obstructive sleep apnoea syndrome which is frequently unrecognized [14,43]. Indeed, the prevalence of obstructive sleep apnoea syndrome is lower in our study than in previous general population studies which have employed more definitive diagnostic methods [11,12]. It seems likely therefore that some patients with obstructive sleep apnoea syndrome will have gone unrecognised altogether [13,14,43] although it is also possible that some may have been misdiagnosed as having another sleep problem, in which case they would have been classified firstly as “any sleep problem” and then as a “sleep problems other than sleep apnoea” in this study. However, previous studies indicate that a diagnosis of obstructive sleep apnoea made by a general practitioner is usually correct [14,44]. A further limitation is that obesity and alcohol which are risk factors for both gout and obstructive sleep apnoea syndrome [25,26] are infrequently coded in primary care databases and hence we were unable to adjust for either of these important potential confounding variables. We acknowledge also that our analysis is likely to have been under-powered to detect a significant association between gout and obstructive sleep apnoea syndrome. However, we believe that this is a novel and interesting potential association which has not been previously studied and for which there is a biologically credible underlying mechanism, namely enhanced degradation of adenosine triphosphate in the presence of hypoxia leading to increased substrate provision for the purine metabolic pathway of which uric acid is the end-product in humans [28-30] and prevalent hyperuricaemia in obstructive sleep apnoea syndrome [19,22-24]. These preliminary findings provide justification for a larger prospective epidemiological study to examine in more detail the possibility that obstructive sleep apnoea syndrome is a risk factor for gout.

We found a significant association between gout and sleep problems other than sleep apnoea. These disorders consisted of a heterogeneous group of conditions, most commonly insomnia and non-specific sleep disorders (data not shown). To our knowledge, associations between these conditions and either gout or hyperuricaemia have not been studied previously and we are not aware of biological mechanisms to link them with either hyperuricaemia or crystal formation. More detailed study of this association, which specific sleep disorders are associated with gout, and the mechanisms underlying this association is warranted.

Notwithstanding the lack of association between gout and obstructive sleep apnoea syndrome after adjustment for confounding factors, our univariate analysis suggests that gout and obstructive sleep apnoea syndrome can co-exist and hence clinicians should be aware of gout as a potential cause of painful joints in patients with obstructive sleep apnoea syndrome and also of the latter as a significant cause of excessive sleepiness in patients with gout. Prompt and accurate recognition of obstructive sleep apnoea syndrome is particularly important because of the associated risk of motor vehicle accidents [45] and legal requirements not to drive unless treated [46]. Furthermore, treatment of obstructive sleep apnoea syndrome with continuous positive airways pressure (CPAP) has been shown to improve certain parameters of the metabolic syndrome such as hypertension [47] and hyperlipidaemia. It is plausible that lowering of uric acid levels with CPAP treatment would theoretically lead to better control of gout although to our knowledge the effect of CPAP on uric acid levels has not been studied and further research is required. Further large prospective epidemiological studies are required to explore this association and establish causality in more detail.

## Conclusions

Our findings support an association between gout and sleep disorders, although the association with obstructive sleep apnoea syndrome does not appear to be independent of co-morbid confounding factors. However, the potential co-existence of these two common conditions is an important message for clinicians and deserves wider recognition. Future studies should focus on establishing the independence of this association and causality and examine whether treatment strategies benefit both conditions.

## Abbreviations

BNF: British National Formulary; CiPCA: Consultations in Primary Care Archive; CPAP: Continuous positive airways pressure; IHD: Ischemic Heart Disease; PiPCA: Prescriptions in Primary Care Archive.

## Competing interests

The authors declare that they have no competing interests.

## Authors’ contributions

ER and CDM conceived the idea for the study. SM performed the statistical analysis. All authors participated in the design of the study, interpretation of data, drafting of the manuscript, and read and approved the final manuscript.

## Authors’ information

ER is a Clinical Senior Lecturer in Rheumatology and Honorary Consultant Rheumatologist. SM is a Research Associate in Clinical Epidemiology. RH is a GP Research Fellow. CDM is a Professor of General Practice.

## Pre-publication history

The pre-publication history for this paper can be accessed here:

http://www.biomedcentral.com/1471-2474/14/119/prepub
